# MYB41, MYB107, and MYC2 promote ABA-mediated primary fatty alcohol accumulation via activation of *AchnFAR* in wound suberization in kiwifruit

**DOI:** 10.1038/s41438-020-0309-1

**Published:** 2020-06-01

**Authors:** Xiaopeng Wei, Linchun Mao, Xiaobo Wei, Ming Xia, Changjie Xu

**Affiliations:** 1 College of Biosystems Engineering and Food Science, Zhejiang Key Laboratory of Agro-Food Processing, Key Laboratory of Agro-Products Postharvest Handling of Ministry of Agriculture and Rural Affairs, Zhejiang University, 310058 Hangzhou, China; 2 Ningbo Research Institute, Zhejiang University, 315100 Ningbo, China; 3 Zhejiang Provincial Key Laboratory of Horticultural Plant Integrative Biology, Zhejiang University, 310058 Hangzhou, China

**Keywords:** Wounding, Wounding, Plant physiology, Plant physiology, Wounding

## Abstract

Wound damage triggers the accumulation of abscisic acid (ABA), which induces the expression of a large number of genes involved in wound suberization in plants. Fatty acyl-CoA reductase (FAR) catalyzes the generation of primary fatty alcohols by the reduction of fatty acids in suberin biosynthesis. However, the regulatory effects of transcription factors (TFs) on *AchnFAR* in response to ABA are unexplored. In this study, kiwifruit *AchnFAR* displayed a biological function analogous to that of FAR in transiently overexpressed tobacco (*Nicotiana benthamiana*) leaves. The positive role of TFs, including AchnMYB41, AchnMYB107, and AchnMYC2, in the regulation of *AchnFAR* was identified. The three TFs could individually bind to the *AchnFAR* promoter to activate gene transcription in yeast one-hybrid and dual-luciferase assays. Transient overexpression of TFs in tobacco leaves resulted in the upregulation of aliphatic synthesis genes (including *FAR*) and the increase in aliphatics, including primary alcohols, α,ω-diacids, ω-hydroxyacids, and fatty acids. Moreover, exogenous ABA treatment elevated TF-mediated *AchnFAR* expression and the accumulation of primary alcohols. Conversely, fluridone, an inhibitor of ABA biosynthesis, suppressed the expression of *AchnFAR* and TF genes and reduced the formation of primary alcohols. The results indicate that AchnMYB41, AchnMYB107, and AchnMYC2 activate *AchnFAR* transcription to promote ABA-mediated primary alcohol formation in wound suberization in kiwifruit.

## Introduction

Plant suberin is an aliphatic and phenolic polymer and is deposited on the inner side of primary cell walls to protect plants against environmental stresses^1^. In terms of aerial tissues, suberin is deposited in fruit skins, bark tissue, and the seed coat^2^. Suberin works as a hydrophobic barrier to restrict the movement of water, solutes, and gases and contributes to the strength of the cell wall^3,4^. Moreover, suberization induced by wounding is confined to an impervious layer of wounded tissue in response to pathogen attack and water loss in potato tubers^5^, tomato fruits^6^, and kiwifruit^7^. Chemically, suberin is a heteropolymer consisting of glycerol, phenolics, and aliphatics impregnated with waxes^8,9^. The predominant aliphatics of suberin include fatty acids, α,ω-diacids, ω-hydroxyacids, and primary alcohols^8,10,11^, which play physiologically important roles in the suberin properties of water sealing and fungal resistance^6,12^. The enzymes required for aliphatic monomer biosynthesis include β-ketoacyl-CoA synthases (KCSs), FARs, fatty acid ω-hydroxylases (CYP86A1 and CYP86B1), and acyl-CoA:glycerol-3-phosphate acyltransferases (GPATs), which have been identified in the model plant species *Arabidopsis*^11^. KCS2 and KCS20 are mainly produced by very-long-chain (≥C20) fatty acids^13,14^. Cytochrome P450 CYP86A1 and CYP86B1 have been reported to generate the specific chain lengths needed for ω-hydroxyacids and α,ω-diacids^15,16^, and GPAT5 and GPAT7 likely catalyze the transfer of a fatty acyl to the *sn*-1 or *sn*-2 position of glycerol 3-phosphate^17–19^. Primary alcohols with C18, C20, C22, and C24 chain lengths are common suberin components in *Arabidopsis*, potato tubers, and tobacco leaves^9,20,21^. FAR reduces fatty acyl-CoA to form primary alcohols in an NADPH-dependent reaction^22,23^. In *Arabidopsis*, the biological function of AtFARs has been identified, and the proteins display distinct substrate specificity in terms of acyl chain length^23–25^. Expression in yeast confirms that AtFAR1, AtFAR4, and AtFAR5 generate primary alcohols with chain lengths in the range of C18 to C22. *AtFAR* mutants indicate that AtFAR1, AtFAR4, and AtFAR5 are most important for the production of C22, C20, and C18 primary alcohols, respectively^25^. Additionally, AtFAR3 is mainly responsible for the production of C24–C28 primary alcohols^24^. However, the gene encoding the FAR protein associated with wound suberization in kiwifruit has not been identified.

Wound damage ultimately induces abscisic acid (ABA), salicylic acid (SA), and jasmonic acid (JA) accumulation, and these plant hormones have therefore been suggested to be mediators of suberization processes^6,26–28^. ABA has been identified as a positive regulator of the gene expression of suberin biosynthesis and the deposition of suberin in kiwifruit^7,29^, tomato fruits^5,30^, potato tubers^6^, and *Arabidopsis* roots^31^. Moreover, the application of fluridone (FLD), which is an effective inhibitor of ABA biosynthesis^32^, has confirmed the role of ABA in the processes associated with wound suberization^6,30,33^. TFs are important components of ABA signaling in response to abiotic stresses in plants. The ABA-dependent signal transduction pathway from abiotic stress perception to gene transcription involves different TFs and their *cis*-acting regulatory elements (ACEs)^34^. MYC and MYB TFs are widely present in plants and have diverse functions^35^. MYCs and MYBs are involved in the ABA-dependent pathway for the upregulation of abiotic stress-responsive genes^34,36^. Characterization of *Arabidopsis* mutants deficient in these MYBs has demonstrated that AtMYB107 acts as a transcriptional activator for suberin monomer biosynthesis^37,38^. AtMYB41 has also been identified as a positive regulator of suberin biosynthesis in transgenic *Arabidopsis* and tobacco overexpression plants^20^. Our previous studies have demonstrated that kiwifruit AchnMYB41 and AchnMYB107 can activate *AchnFHT* and *AchnCYP86A1*, which are involved in ABA-mediated wound suberization^39,40^, while AchnMYC2 has been identified as an activator of *AchnCYP86A1*^40^. Therefore, it is speculated that AchnMYB41, AchnMYB107, and AchnMYC2 could act as activators of *AchnFAR* in ABA-mediated wound suberization in kiwifruit.

In this study, *AchnFAR*, *AchnMYB41*, *AchnMYB107*, and *AchnMYC2* were cloned from kiwifruit. The biological function of AchnFAR as a FAR was demonstrated in transiently overexpressed tobacco leaves. The regulatory function of AchnMYB41, AchnMYB107, and AchnMYC2 on *AchnFAR* was demonstrated with yeast one-hybrid and dual-luciferase assays together with transient overexpression in tobacco leaves. Moreover, the gene expression and primary alcohol formation induced by exogenous ABA in wounded tissues of kiwifruit were determined.

## Results

### Gene isolation and analysis

**Fig. 1:**
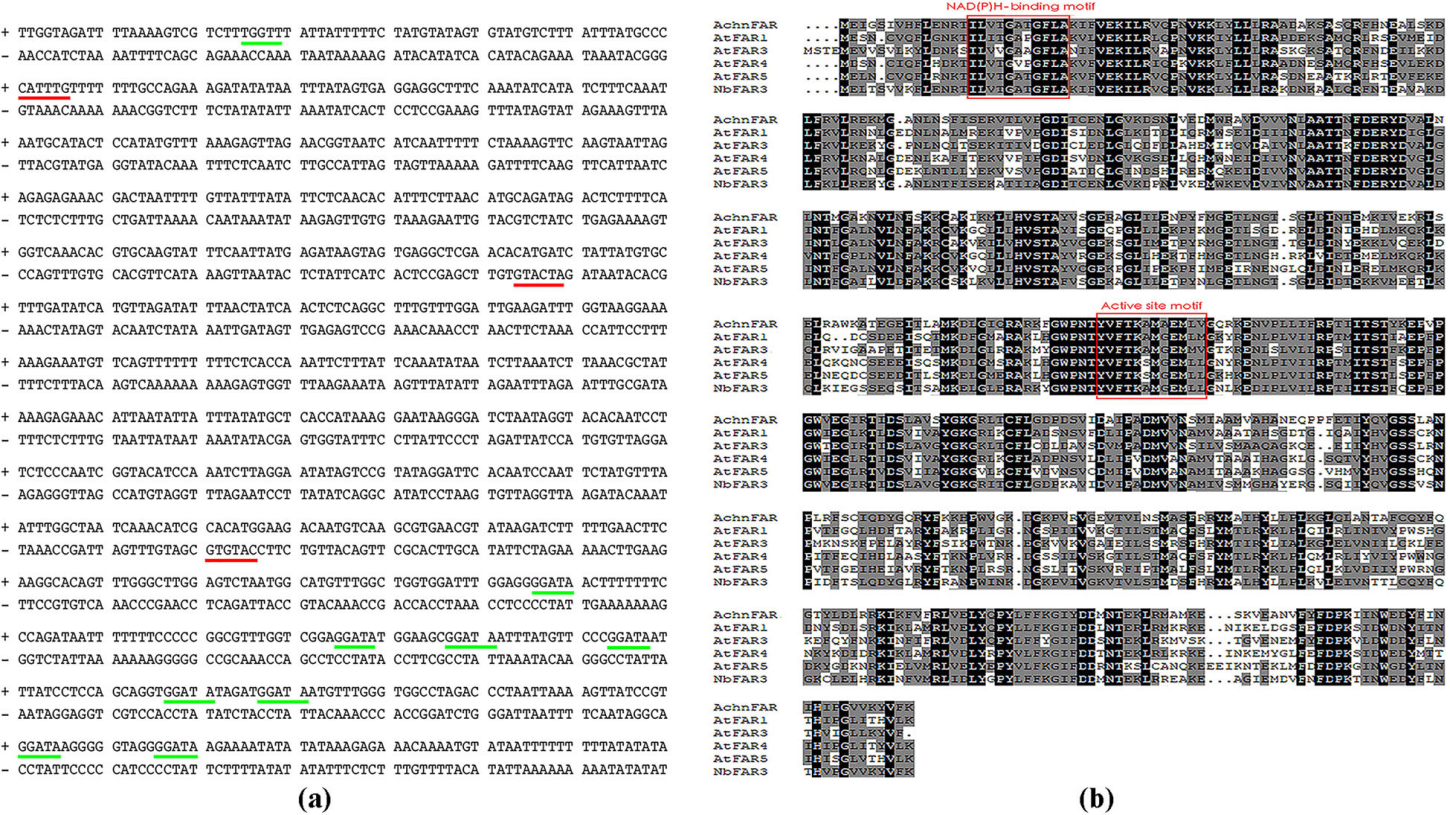
Sequence analysis of *AchnFAR*. **a** The *cis*-acting regulatory elements of the AchnFAR promoter. The red and green lines indicate MYCRE and MYBRE, respectively. **b** The amino acid sequences of AchnFAR, AtFAR1, AtFAR3, AtFAR4, AtFAR5, and NbFAR3 were aligned using ClustalX.

The full-length sequence of *AchnFAR* was obtained using the sequences of AtFAR1, AtFAR3, AtFAR4, and AtFAR5 as queries against the kiwifruit genome database with tblastx analysis. The expression levels of *AchnFAR* were analyzed in different tissues with quantitative reverse transcription-polymerase chain reaction (qRT-PCR, Fig. S1). A higher level of *AchnFAR* transcripts was detected in the fruits compared to the roots, shoots, and leaves. A fragment (1506 bp) of the *AchnFAR* promoter was cloned from kiwifruit DNA. Analysis of the ACE of the promoter revealed three MYC recognition elements (MYCRE, red line) and nine MYB recognition elements (MYBRE, green line) using the PlantCARE and PLACE databases (Fig. 1a). The full-length CDS of *AchnFAR* was 1479 bp in length and encoded a protein of 492 amino acids with a deduced molecular mass 55.91 kDa. AchnFAR shared the highest sequence identity with NbFAR3 (71%), followed by AtFAR3 (60%), AtFAR1 (56%), AtFAR4 (56%), and AtFAR5 (54%). The protein shared two conserved regions, including an NAD(P)H-binding site motif and a classic active site motif^23^ (Fig. 1b). Phylogenic analysis of the 21 FARs revealed that AchnFAR closely grouped together with AtFARs, including AtFAR3, AtFAR1, AtFAR4, and AtFAR5 (Fig. S2), which are involved in primary alcohol formation for suberin^25^, suggesting that AchnFAR has an analogous function in suberin primary alcohol biosynthesis in kiwifruit.

**Fig. 2:**
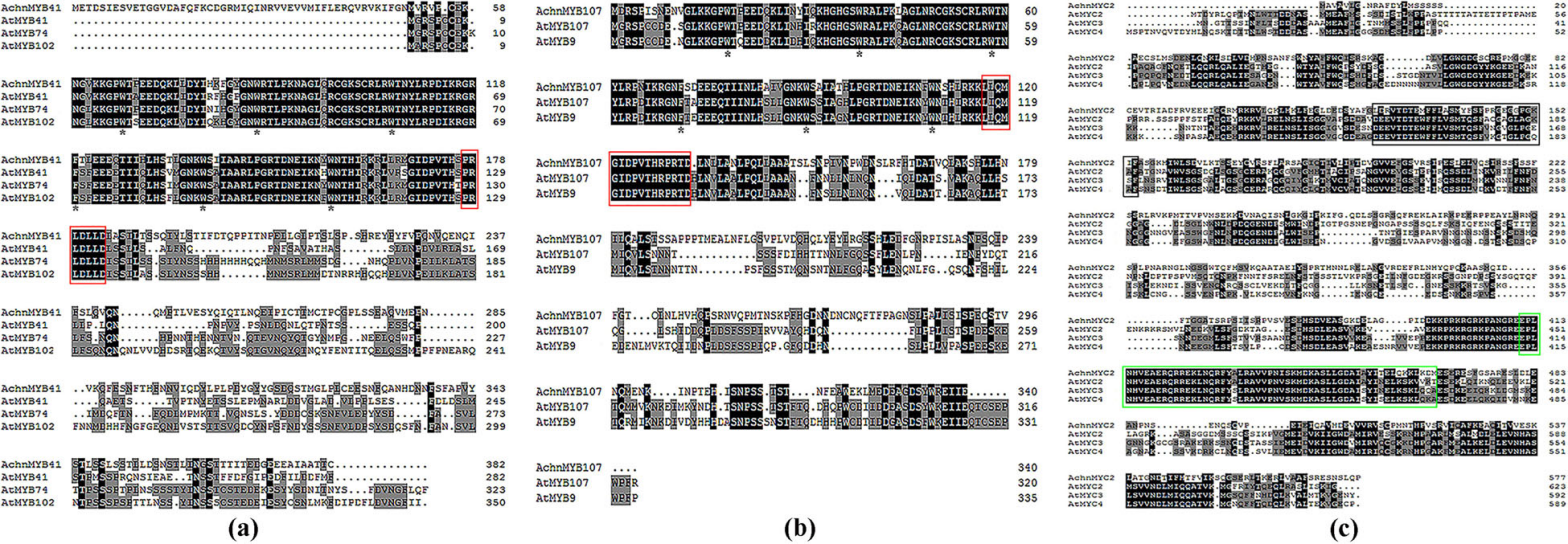
Sequence analyses of MYB41, MYB107, and MYC2. **a**–**c** Alignment analysis of AchnMYB41, AchnMYB107, and AchnMYC2. The arrowheads and red boxes indicate the R2R3-MYB domain and the conserved motif of MYB41 and MYB107, respectively. The green box indicates the bHLH domain, and the acidic region corresponding to the MYC2 activation domain is indicated in the black box. The alignments were performed using ClustalX.

The full-length sequences of the *AchnMYB*s and *AchnMYC*s were obtained using the sequences of AtMYB41, AtMYB107, and AtMYC2 as queries against the kiwifruit genome database via tblastx analysis. A yeast one-hybrid assay was used to screen the interacting TFs using the *AchnFAR* promoter as bait (Fig. S3). Of the AchnMYBs and AchnMYCs, which included Achn310341, Achn313181, Achn313331, Achn318681, Achn173251, Achn136071, Achn031311, Achn084621, AchnMYB41, AchnMYB107, and AchnMYC2, AchnMYB41, AchnMYB107, and AchnMYC2 could bind to the *AchnFAR* promoter. Therefore, AchnMYB41, AchnMYB107, and AchnMYC2 were used for further functional identification. According to the phylogenetic tree, AchnMYB41 and AchnMYB107 clustered with AtMYB41 and AtMYB107, respectively (Fig. S4a); AchnMYC2 closely clustered with AtMYC2, AtMYC3, and AtMYC4 (Fig. S4b). Analysis of the MYB and MYC proteins showed that the primary structure of two AchnMYBs had an R2R3-MYB domain at the N-terminus (Fig. 2a, b, asterisks), followed by conserved motifs (red box)^41^, while AchnMYC2 had an acidic region corresponding to the MYC2 activation domain (Fig. 2c, black box) and contained a DNA-binding motif of the bHLH domain (green box)^42^.

### Production of primary alcohols associated with *AchnFAR*

**Fig. 3:**
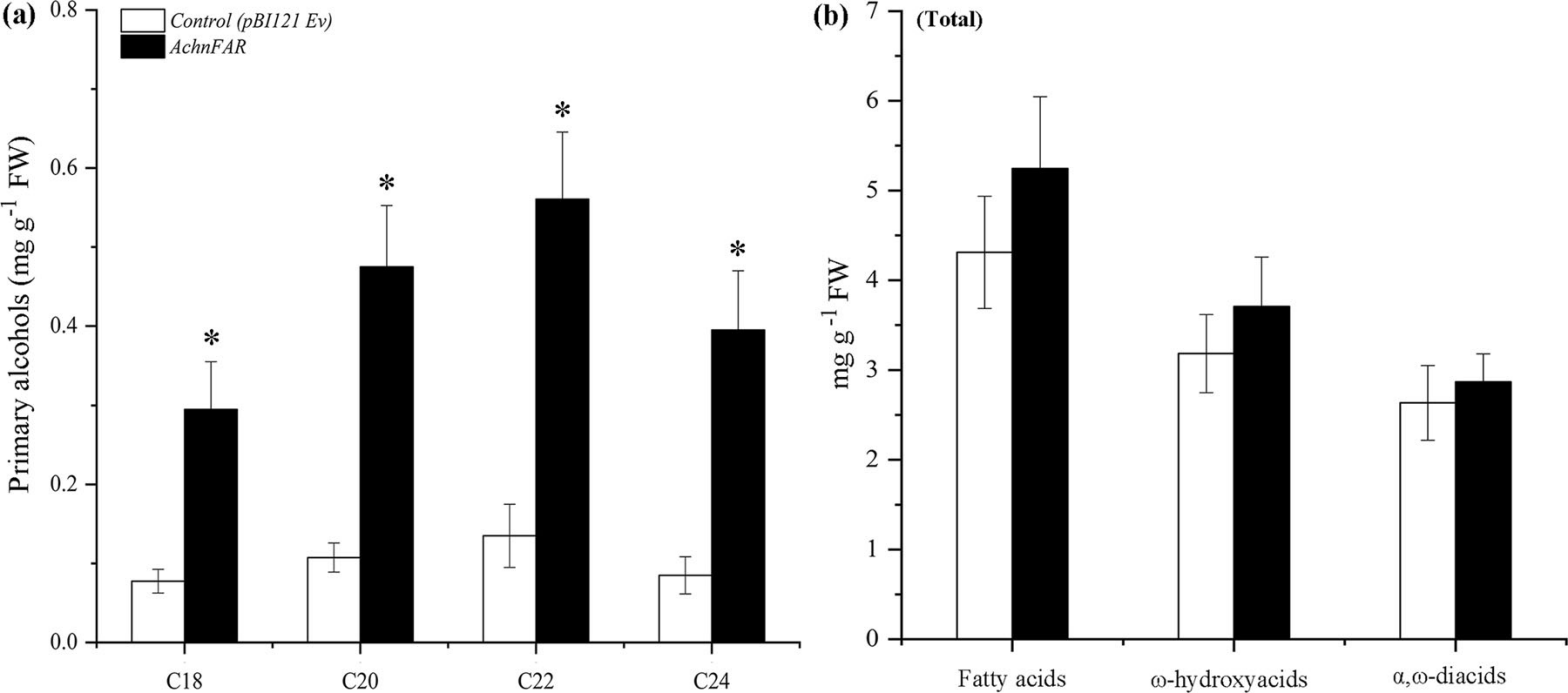
Aliphatic composition in control and *AchnFAR*-infiltrated tobacco leaves. **a** All primary alcohol monomers identified. **b** Total amounts of fatty acids, ω-hydroxyacids, and α,ω-diacids compounds. The error bars indicate the SDs of three independent experiments. The asterisks represent significant differences compared with the control using a *t*-test (*p < 0.05).

The biological function of AchnFAR was identified by transient overexpression of *AchnFAR* in tobacco leaves with the determination of suberin monomer (Fig. 3a). Inspection of the primary alcohol subclass in the overexpression leaves revealed a significant increase relative to that of the control. The primary alcohols with chain lengths of C20–C24 increased by 4.1–4.6-fold compared with those of the control, while the amount of C18 primary alcohol increased by 3.8-fold. Additionally, the quantities of α,ω-diacids, ω-hydroxyacids, and fatty acids were also measured (Fig. 3b). The total amounts of these lipid masses in the overexpression leaves exhibited a slight increase, but there were no significant differences in *AchnFAR*-overexpressing leaves and the controls.

### Activation of AchnMYB41, AchnMYB107, and AchnMYC2 on the promoter of *AchnFAR*

**Fig. 4:**
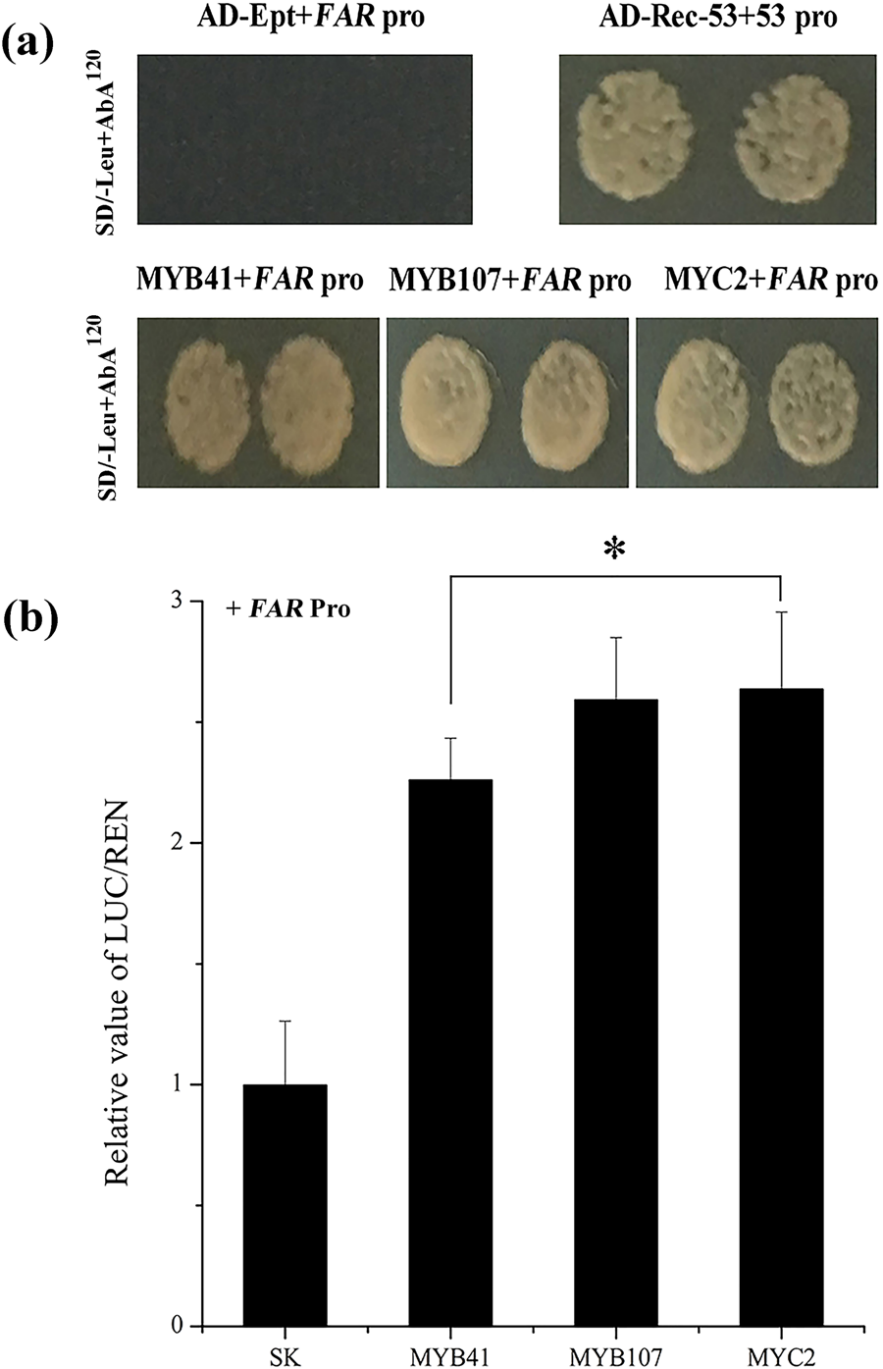
Positive regulation of AchnMYB41, AchnMYB107, and MYC2 on the *AchnFAR* promoter. **a** AchnMYB41, AchnMYB107, and AchnMYC2 individually bound to the *AchnFAR* promoter according to the yeast one-hybrid assay. p53-AbAi with AD-Rec-p53 was the positive control. *AchnFAR*-AbAi with an AD-empty vector was used as a negative control. **b** AchnMYB41, AchnMYB107, and AchnMYC2 activated the *AchnFAR* promoter according to the dual-luciferase assays. The LUC/REN of the SK with the promoter was set as 1. Ept, empty; Pro, promoter. The error bars indicate the SDs of three independent experiments. The asterisks represent significant differences relative to the control using a *t*-test (*p < 0.05).

Based on the PlantCARE and PLACE databases, the *AchnFAR* promoter contained MYBREs and MYCREs (Fig. 1a). A yeast one-hybrid assay was thus used to confirm whether AchnMYB41, AchnMYB107, and AchnMYC2 could bind to the *AchnFAR* promoter *in vitro*. The results showed that AchnMYB41, AchnMYB107, and AchnMYC2 could individually interact with the *AchnFAR* promoter (Fig. 4a). Dual-luciferase assays of tobacco leaves were further used to assess whether these interactions could drive *AchnFAR* expression *in vivo* (Fig. 4b). The results showed that AchnMYB41, AchnMYB107, and AchnMYC2 significantly activated the *AchnFAR* promoter, causing 2.3-, 2.6-, and 2.6-fold enhancement, respectively.

### *AchnMYB41*, *AchnMYB107*, and *AchnMYC2* promotion of gene expression and monomer formation of suberin

**Fig. 5:**
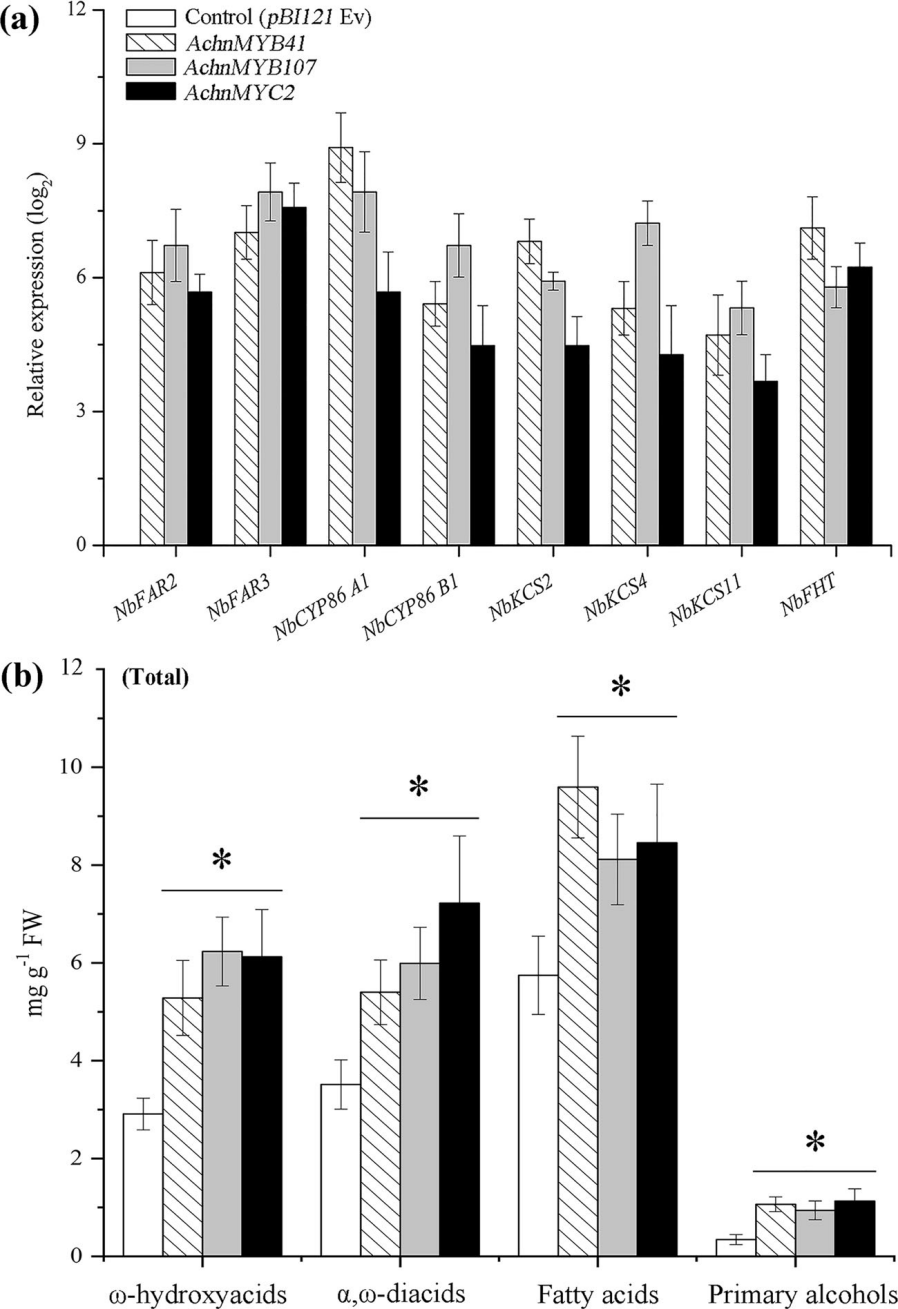
Relative expression change (log_2_) of suberin biosynthetic genes and aliphatic composition in *AchnMYB*_*41*_-, *AchnMYB*_*107*_-, and *AchnMYC*_*2*_-infiltrated tobacco leaves compared with control leaves. **a** Expression of genes in tobacco leaves overexpressing transcription factors compared with control leaves. **b** Total contents of ω-hydroxyacids, α,ω-diacids, fatty acids, and primary alcohol compounds. The error bars indicate the SDs of three independent experiments. The asterisks represent significant differences relative to the *t*-test (*p < 0.05).

The *AchnMYB41*, *AchnMYB107*, and *AchnMYC2* activation effects on the genes of suberin biosynthesis were further confirmed in transiently overexpressed tobacco leaves (Fig. 5). The expression of genes involved in the aliphatic pathway was strongly increased by the overexpression of the TFs (Fig. 5a). The expression of the tobacco homologs of *AchnFAR*, *FAR2*, and *FAR3* was notably increased by the overexpression of *AchnMYB41*, *AchnMYB107*, or *AchnMYC2*, respectively. In particular, the expression level of *FAR3* increased by 7.0-, 7.9-, and 7.6-fold compared to that of the control, respectively. These sharp increases in gene expression were accompanied by 3.1-, 2.7-, and 3.3-fold increases in the total amounts of primary alcohols (Fig. 5b). *CYP86A1* and *CYP86B1*, which encode long-chain and very-long-chain fatty acid ω-hydroxylases, which are responsible for generating the corresponding ω-hydroxyacids, and α,ω-diacids^15,16^ were also notably elevated. Consistent with the gene expression data, the total contents of ω-hydroxyacids and α,ω-diacids notably increased in response to the overexpression of the three TFs. KCS proteins are the key enzymes for the production of very-long-chain fatty acids in suberin biosynthesis^13,14^. *KCS2*, *KCS4*, and *KCS11* were significantly upregulated in response to the overexpression of the three TFs. Likewise, compared with those in the control leaves, the total amounts of fatty acids in the *AchnMYB41-*, *AchnMYB107-*, and *AchnMYC2*-overexpressing leaves increased by 1.6-, 1.4-, and 1.5-fold, respectively. Moreover, the *FHT* gene, which encoded feruloyl transferase, which transfers a feruloyl moiety to ω-hydroxyacids and primary alcohols to cross-link suberin aliphatic and phenolic domains^43,44^, was also significantly induced in the overexpression leaves.

### Increase in gene expression and primary alcohol formation in response to exogenous ABA

**Fig. 6:**
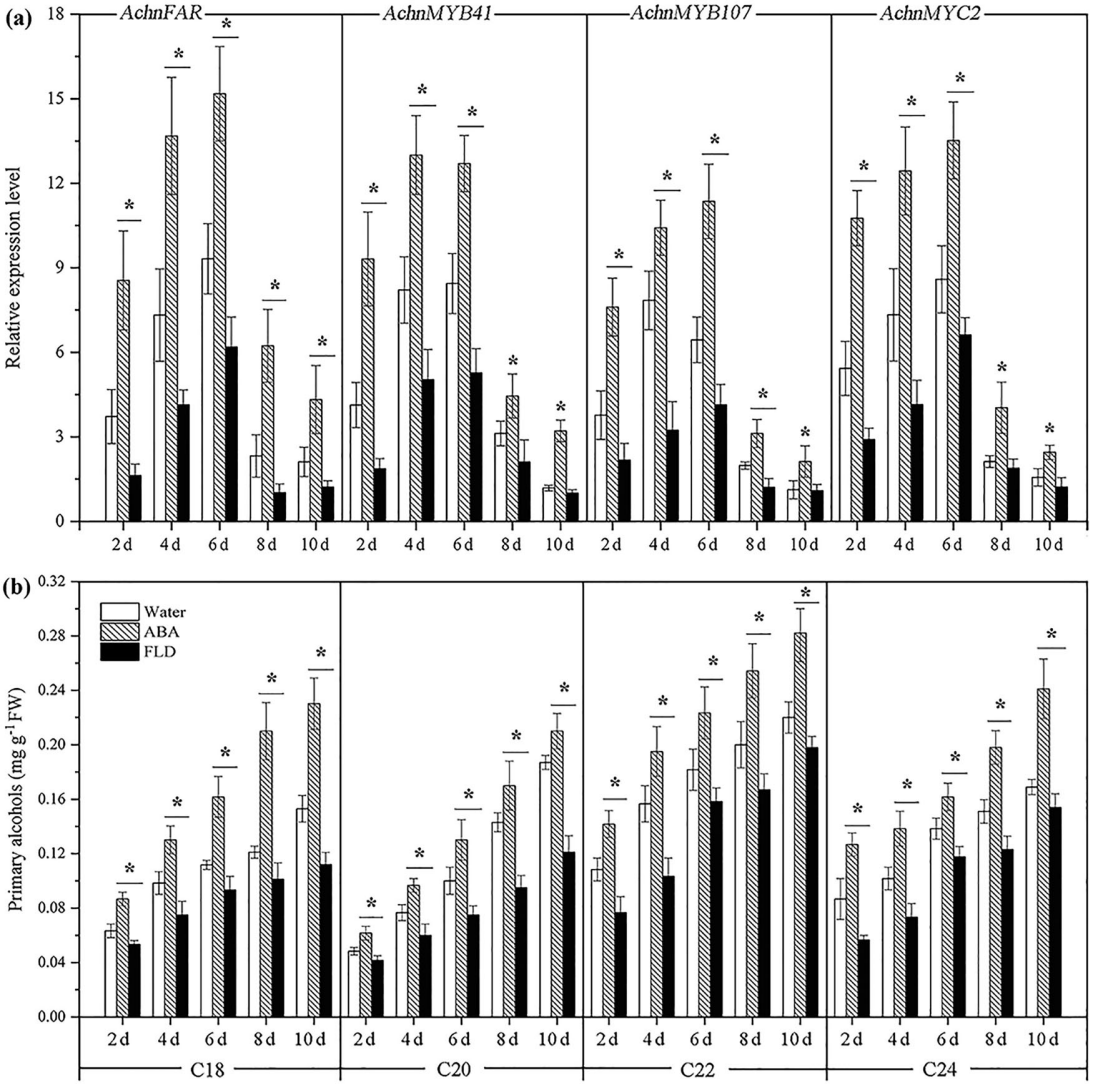
Gene expression and the amounts of primary alcohols in ABA-, FLD-, and water-treated kiwifruit tissues. **a** Expression levels of *AchnFAR*, *AchnMYB41*, *AchnMYB107*, and *AchnMYC2*. The gene expression is presented as the fold change compared with the initial value upon wounding. **b** Contents of primary alcohols. The error bars indicate the SDs of three independent experiments. The asterisks represent significant differences relative to the control using a *t*-test (*p < 0.05).

The expression levels of *AchnFAR*, *AchnMYB41*, *AchnMYB107*, and *AchnMYC2* in water-, ABA-, and FLD-treated wound tissues of kiwifruit were measured with qRT-PCR (Fig. 6a). The expression level of *AchnFAR* in all treatments rapidly increased, peaked at 6 days after wounding, and then declined. Exogenous ABA significantly induced *AchnFAR* expression; however, FLD suppressed gene expression. At 6 days after wounding, *AchnFAR* expression in ABA-treated tissues was 1.6-fold higher after water treatment and 2.5-fold higher after FLD treatment. Likewise, the expression of *AchnMYB41*, *AchnMYB107*, and *AchnMYC2* also notably increased in response to exogenous ABA but inhibited by FLD treatment.

Since AchnFAR shares high homology with AtFARs that produce primary alcohols associated with suberin biosynthesis in *Arabidopsis*, the contents of primary alcohols in flesh tissues of nonwounded kiwifruit and in water-, ABA-, and FLD-treated tissues were analyzed. To determine the contents of primary alcohols, samples were subjected to depolymerization and analyzed with gas chromatography–mass spectrometry. Primary alcohols with chain lengths of C18–C24 were detected in the kiwifruit, and the content of C22 primary alcohols was highest, followed by the contents of C24, C18, and C20 primary alcohols (Fig. S5). In wounded tissues of kiwifruit, ABA notably increased the accumulation of primary alcohols with chain lengths of C18–C24, while FLD notably reduced alcohol formation during the 10 days period after wounding (Fig. 6b).

## Discussion

Primary alcohols are important components of aliphatics, which have a crucial role in suberin permeability characteristics^45^ and suberin assembly^8^. Primary alcohols are provided as substrates for fatty alcohol caffeoyl-CoA caffeoyl transferase (FACT) and feruloyl transferase, which transfer caffeoyl and feruloyl to primary alcohols, respectively, to cross-link the aliphatic and phenolic domains for suberin assembly^41,44,46^. In *Arabidopsis*, AtFAR1, AtFAR4, and AtFAR5 generate C22, C20, and C18 primary alcohols, respectively, in association with suberization, while AtFAR3 produces C24–C28 primary alcohols. In the present study, the AchnFAR protein shared 54–60% identity with NADPH-dependent membrane-associated alcohol-forming AtFARs and had the same length (Fig. 1b). Transient overexpression of *AchnFAR* significantly increased the amount of C18–C24 primary alcohols in tobacco leaves, especially the C20–C24 alcohols, while the total amounts of ω-hydroxyacids, fatty acids, and α,ω-diacids showed no significant differences (Fig. 3). In kiwifruit, the expression of *AchnFAR* in all the treatments increased for 6 days after wounding but then declined (Fig. 6). Gene expression resulted in the accumulation of primary alcohols (Fig. 6). At 6 days after wounding, the ABA contents in all the tissues probably decreased^47^, which resulted in a decrease in *AchnFAR* expression. Additionally, aliphatic formation for suberin deposition was probably complete at 6 days after wounding^29,48^, and therefore, the negative regulation of transcriptional repressors could inhibit *AchnFAR* expression to prevent the accumulation of aliphatics^39,49^. These results suggest that *AchnFAR*, which encodes a FAR, generates primary alcohols involved in wound suberization in kiwifruit.

It is well known that several MYBs positively regulate suberin biosynthesis. In *Arabidopsis* and apple, suberin biosynthetic genes, including *FAR*, *CYP86A1*, and *KCS*, and suberin deposition are significantly induced in response to the overexpression of *AtMYB41* and *MdMYB93*^20,21^. AtMYB9 and AtMYB107 act as positive regulators in suberin biosynthesis^37,38^. AtMYC2 can bind to a recognition element (CACATG) within the *RD22* promoter to positively regulate the gene^36^. TFs usually show sequence-specific DNA binding and are capable of regulating gene transcription^50^. In this study, AchnMYB41, AchnMYB107, and AchnMYC2 shared the R2R3-MYB and bHLH domains, both of which are considered DNA-binding domains (Fig. 2). Based on the ACE analysis, the promoter of *AchnFAR* contained a MYBRE and MYC2RE (CACATG) (Fig. 1a). Hence, AchnMYB41, AchnMYB107, and AchnMYC2 could possibly regulate *AchnFAR* by interacting with the gene promoter. Yeast one-hybrid and dual-luciferase assays further confirmed that the three TFs could directly bind to the *AchnFAR* promoter and activate gene transcription (Fig. 4). Furthermore, the positive role of TFs such including AchnMYB41, AchnMYB107, and AchnMYC2 in the regulation of *AchnFAR* was demonstrated in transiently overexpressed tobacco leaves. Overexpression of the TFs individually triggered the expression of aliphatic biosynthetic genes, including *FAR*, *CYP86A1*, *CYP86B1*, and *KCS* (Fig. 5a), which are involved in the reduction, ω-hydroxylation, and elongation of fatty acids. This activation of aliphatic genes was supported by the increases in the total amounts of suberin components, including fatty acids, primary alcohols, ω-hydroxyacids, and α,ω-diacids (Fig. 5b). KCS must provide substrates for FAR, CYP86A1, and CYP86B1 to produce primary alcohols, α,ω-diacids, and ω-hydroxyacids, respectively. Collectively, the results suggest that AchnMYB41, AchnMYB107, and AchnMYC2 work as activators of *AchnFAR* and probably other genes involved in aliphatic compounds to coordinate the biosynthesis of aliphatic monomers.

**Fig. 7:**
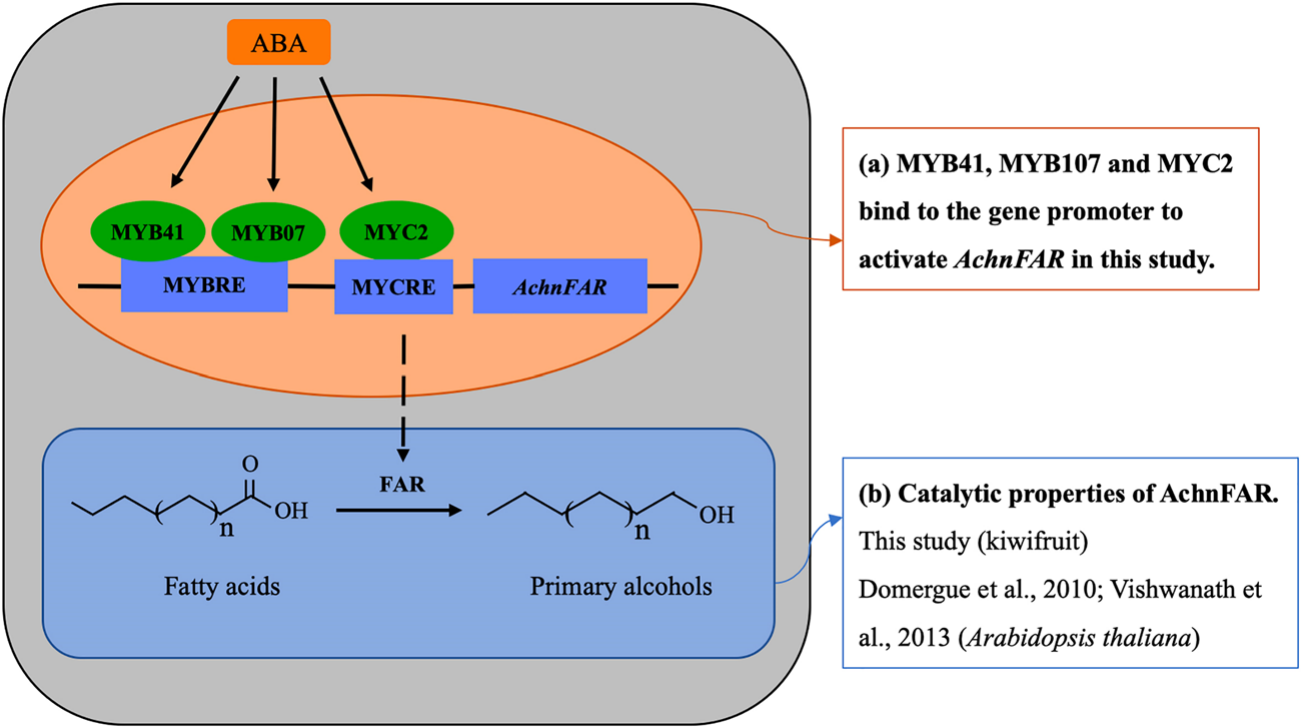
Model for the ABA-responsive expression of the *AchnFAR* gene via MYB and MYC recognition elements. ABA induces AchnMYB41, AchnMYB107, and AchnMYC2, which activate *AchnFAR*, which is involved in primary alcohol biosynthesis.

The mechanisms underlying ABA responses have been characterized in many studies in which a large number of TFs in the molecular network linking the ABA signal to the stress responses have been identified^51^. Previous studies have indicated that MYBs and MYC2 work as transcriptional activators in the ABA signaling pathway under stress conditions in plants^20,34,36,52^. In this study, the expression of *AchnFAR* notably increased in response to exogenous ABA (Fig. 6), and the *AchnFAR* promoter was individually activated by AchnMYB41, AchnMYB107, and AchnMYC2 (Figs. 4 and 5). Additionally, *AchnMYB41*, *AchnMYB107*, and *AchnMYC2* notably increased in response to exogenous ABA but were inhibited by FLD (Fig. 6). Hence, it could be speculated that *AchnMYB41*, *AchnMYB107*, and *AchnMYC2* follow an ABA-dependent signal transduction pathway to activate *AchnFAR*, which is involved in the production of primary alcohols (Fig. 7). *AchnMYB41*, *AchnMYB107*, and *AchnMYC2* are induced by ABA, and then the induced TFs individually bind to the *AchnFAR* promoter to activate gene transcription (Fig. 7a). Afterward, AchnFAR reduces fatty acids that form primary alcohols (Fig. 7b). This study provides a mechanism by which AchnMYB41, AchnMYB107, and AchnMYC2 regulate the activity of *AchnFAR* in response to ABA.

## Materials and methods

### Materials and treatments

Kiwifruit (*Actinidia chinensis* Planch cv. Xuxiang) were harvested in Hangzhou City, Zhejiang Province, China. Fruit surface sterilization and treatments were conducted as described by Wei et al.^39^. Briefly, kiwifruit was sterilized using NaClO solution for 5 min. The fruit was then wounded by being cut longitudinally into halves that were treated with ABA, FLD, and sterile water through vacuum infiltration. The treated halves were put into a sterile incubator for wound healing in the dark at 20 °C. The samples were collected at the indicated time points and frozen in liquid nitrogen for further analysis. The fruits, shoots, roots, and leaves were harvested for *AchnFAR* expression analysis.

### Yeast one-hybrid assays

Yeast one-hybrid assays were performed in accordance with the methods of Wei et al.^39^. The promoter of *AchnFAR* was cloned into a pAbAi vector. To generate bait-reporter Y1HGold strains, AchnFAR-AbAi and p53-AbAi were individually linearized for transformation. The Y1HGold strains were subsequently inoculated onto plates that contained SD/-Ura supplemented with 0, 50, 100, 120, 150, and 200 ng ml^−1^ aureobasidin A (AbA) for the autoactivation test. *AchnMYB41*-CDS, *AchnMYB107*-CDS, and *AchnMYC2*-CDS were inserted into an AD (pGADT7) vector, after which the vector was then transformed into Y1HGold strains that were inoculated on SD plates lacking leucine but supplemented with 120 ng ml^−1^ AbA (SD/−Leu+AbA^120^) to test the interactions. The primers used for the yeast one-hybrid assays are presented in Table S1.

### Dual-luciferase assays

Dual-luciferase assays were performed in accordance with the methods of Wei et al.^39^. In brief, *AchnMYB41*-CDS, *AchnMYB107*-CDS, and *AchnMYC2*-CDS were individually cloned into SK (pGreen II 0029 62-SK), while the *AchnFAR* promoter was cloned into pGreen II 0800-LUC. *Agrobacterium* (GV3101) was used for plasmid transformation, which was infiltrated into tobacco (*Nicotiana benthamiana*) leaves. A dual-luciferase reporter assay system was used to detect firefly luciferase (FLUC) and Renilla luciferase (RLUC) activities. The primers used for dual-luciferase assay are presented in Table S1.

### Transient overexpression

To investigate the function of *AchnMYB41*, *AchnMYB107*, *AchnMYC2*, and *AchnFAR* in suberin biosynthesis, transient overexpression analyses were conducted in tobacco (*N. benthamiana*)^40^. The full-length *AchnMYB41*-CDS, *AchnMYB107*-CDS, *AchnMYC2*-CDS, and *AchnFAR*-CDS were inserted into pBI121 in place of the GUS gene. *Agrobacterium* cells (EHA105) were used for plasmid transformation and then infiltrated into tobacco leaves. pBI121 (empty vectors) was transformed into EHA105 and infiltrated into tobacco leaves as a control. Transient overexpression was repeated in three independent experiments. Six days after infiltration, the tobacco leaves were used for qRT-PCR and suberin determination. The primers used for transient overexpression are presented in Table S1.

### RNA isolation and qRT-PCR

Total RNA was isolated from kiwifruit samples and tobacco leaves according to the CTAB method^53^. A NanoDrop 2000 instrument (NanoDrop Technologies, Inc., USA) was used to investigate the quality of the RNA samples. cDNA synthesis and qRT-PCR were performed as described by Wei et al.^40^. qRT-PCR was conducted with a Biosystems 7500 real-time PCR system (Thermo Scientific, Inc., USA). Each primer pair without a template was used as a negative control. The 2^−ΔΔCt^ method was used to calculate kiwifruit and tobacco gene expression using *AchnActin* and *Nbβ-Tubulin* as internal standards. The analyses were conducted for three independent experiments. The primers used for qRT-PCR are presented in Table S1.

### Suberin monomer analysis

Suberin was depolymerized using acid-catalyzed methanolysis^21,54^. Briefly, samples of kiwifruit and tobacco were added to glass tubes with screw caps. CH_3_OH/H_2_SO_4_ (20:1, v/v) was subsequently added, after which the mixture was incubated in a water bath for 3 h at 85 °C. Afterward, dichloromethane and sodium chloride solution were added. The mixtures were washed with distilled water, and anhydrous Na_2_SO_4_ was added to the organic phase. After centrifugation, the solvent was dried by evaporation. The residues were trimethylsilyl derivatized according to the methods of Wei et al.^40^. The dried derivatives were dissolved in 0.5 ml of CH_2_Cl_2_ for further analysis using a GC–MSD (7890B-5977A) system^29^. The analysis was performed for three independent replicates.

## Supplementary information

Figure S1

Figure S2

Figure S3

Figure S4

Figure S5

Table S1

Table S2
