# Supplementary figures and images for: MYB41, MYB107, and MYC2 promote ABA-mediated primary fatty alcohol accumulation via activation of AchnFAR in wound suberization in kiwifruit
Source: Hortic Res. 2020 Jun 1;7:86. doi: 10.1038/s41438-020-0309-1 (PMC7261769; doi:10.1038/s41438-020-0309-1)

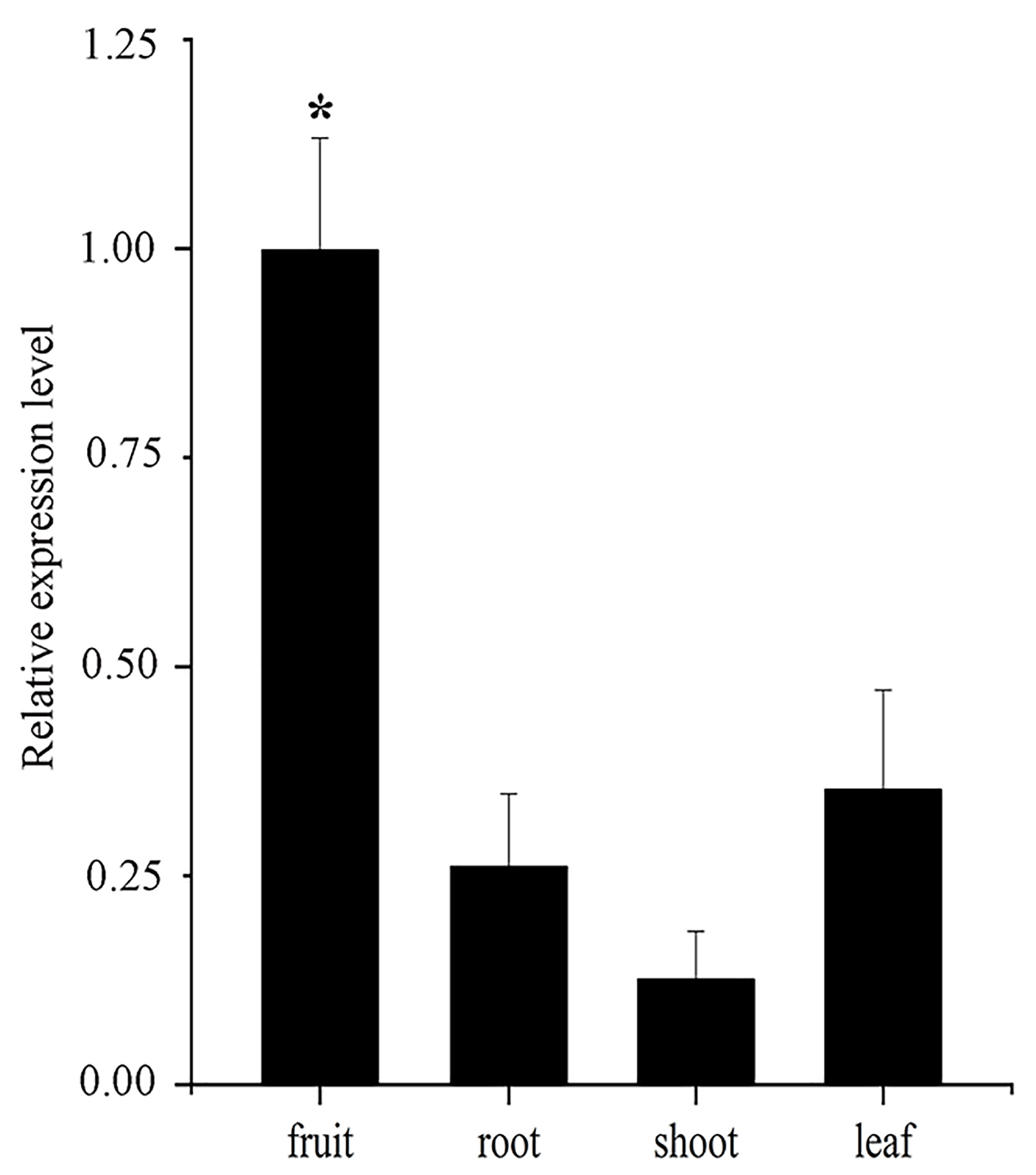

Supplement: Supplementary file 1 — Figure S1 [file 41438_2020_309_MOESM1_ESM.jpg]

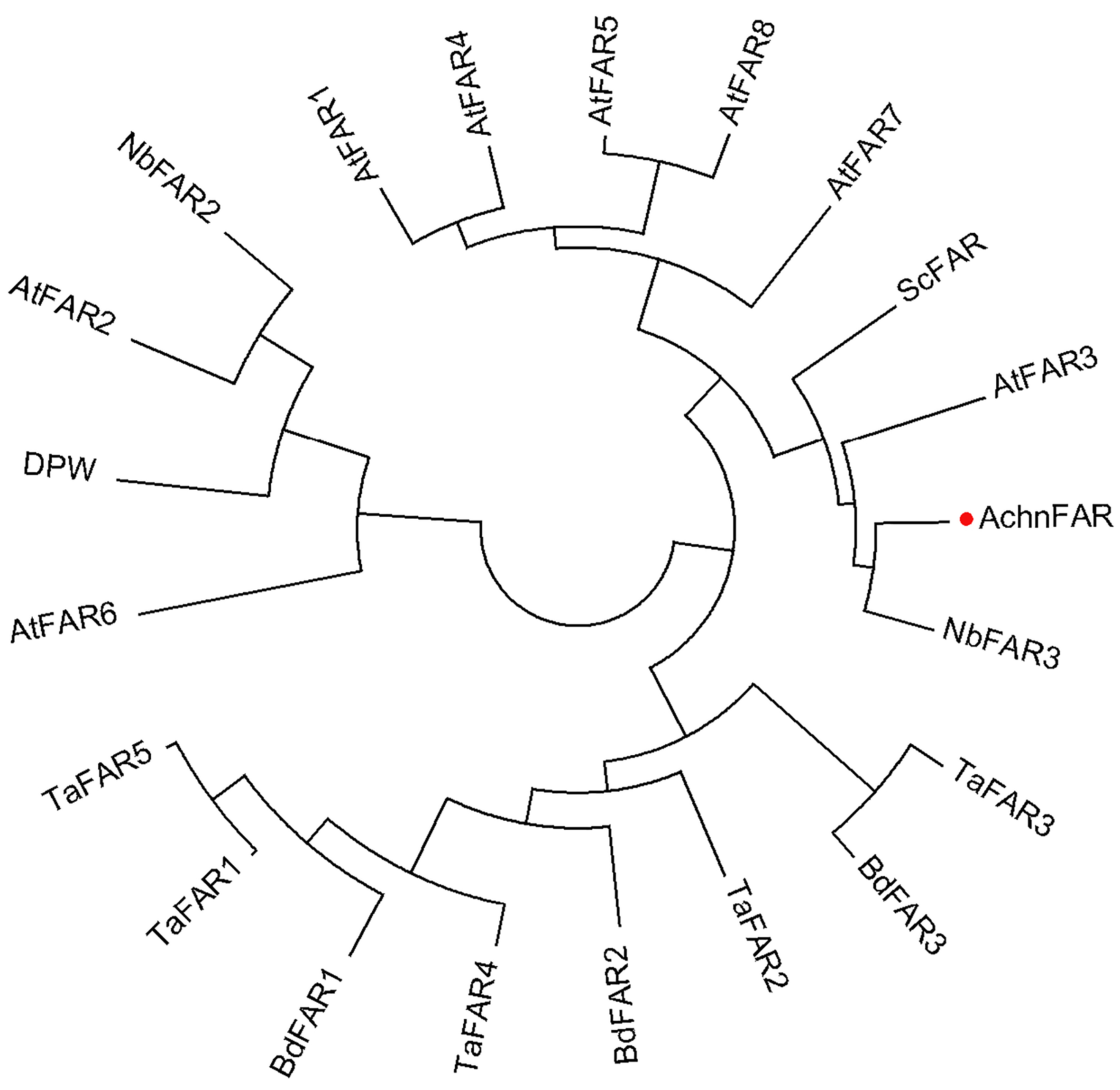

Supplement: Supplementary file 2 — Figure S2 [file 41438_2020_309_MOESM2_ESM.jpg]

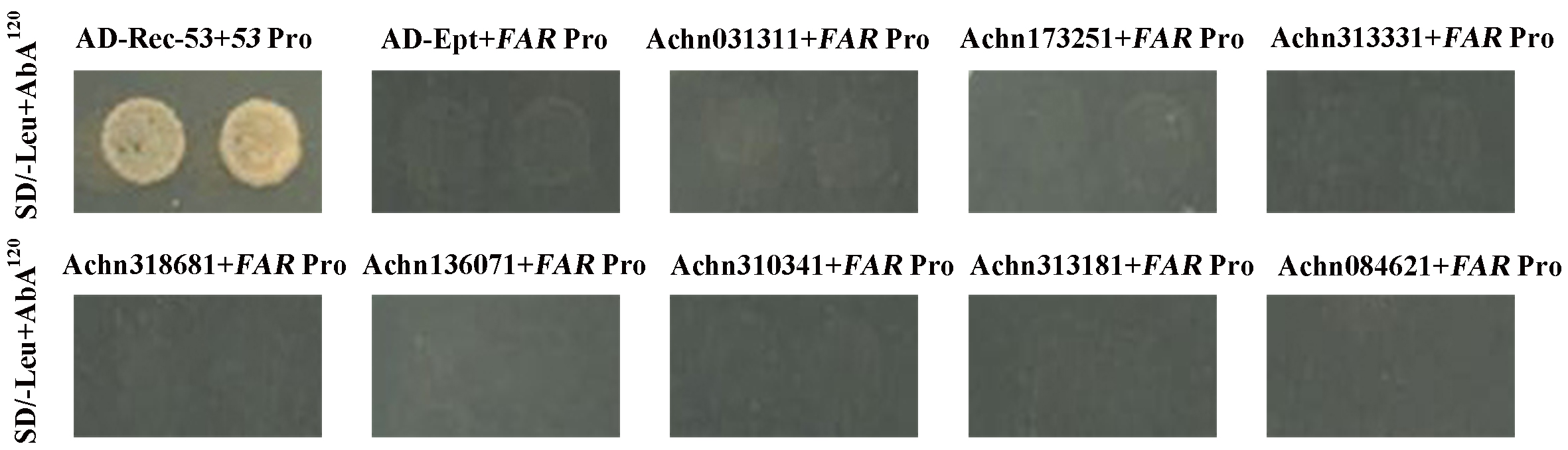

Supplement: Supplementary file 3 — Figure S3 [file 41438_2020_309_MOESM3_ESM.jpg]

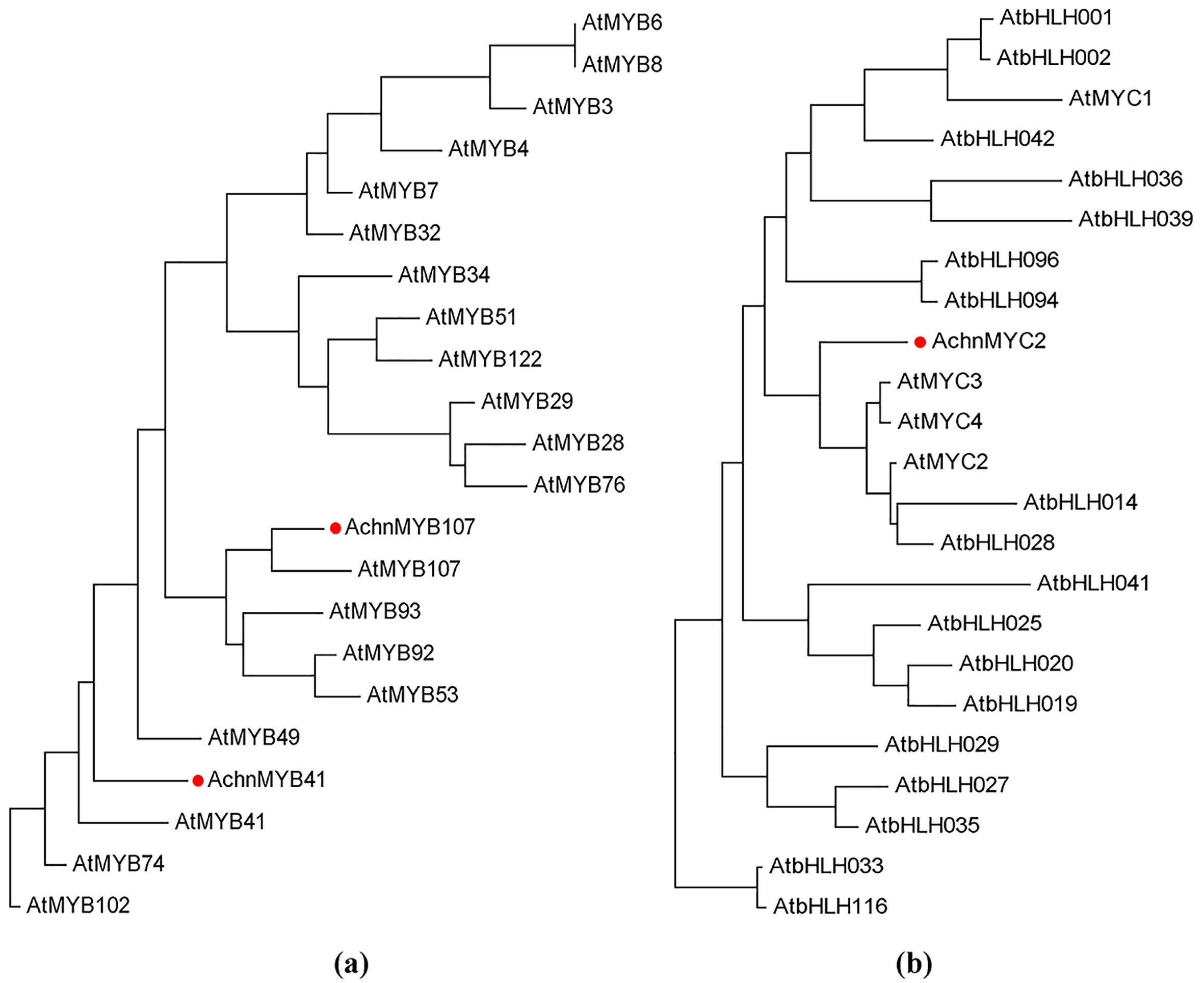

Supplement: Supplementary file 4 — Figure S4 [file 41438_2020_309_MOESM4_ESM.jpg]

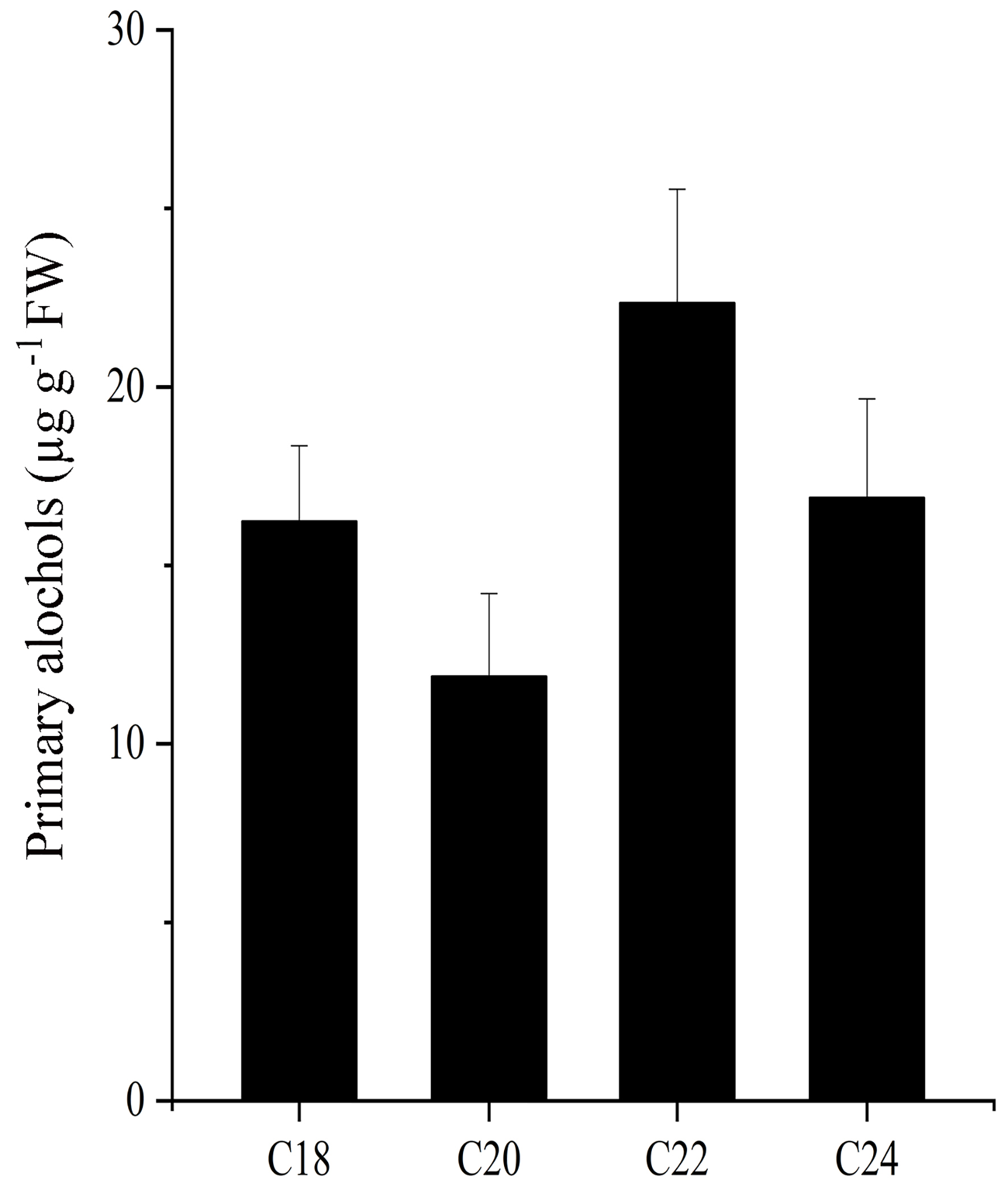

Supplement: Supplementary file 5 — Figure S5 [file 41438_2020_309_MOESM5_ESM.jpg]
